# Sensory and Consumer Research Has a Role in Supporting Sustainability of the Food System

**DOI:** 10.3390/foods11131958

**Published:** 2022-07-01

**Authors:** Antti Knaapila

**Affiliations:** Department of Food and Nutrition, University of Helsinki, 00014 Helsinki, Finland; antti.knaapila@helsinki.fi

How can sensory and consumer research contribute to the sustainability of a food system? This question was discussed in this Special Issue of *Foods* with its 20 articles, including 18 original research articles and 2 reviews. These articles showcase recent sensory and consumer research on the topic in a versatile way. We learn what kind of food ingredients and products have been of interest, which questions have been studied, which methods have been applied, and what were the results and conclusions. The contributions by more than 90 authors show not only progress in the field but also propose future directions.

The sustainability of a food system can be improved by many ways. A variety of approaches were investigated in the articles of this issue. One of the main topics was the sensory quality and consumer responses to novel alternatives to conventional animal-based foods. For example, plant-based meat and dairy analogues may help omnivores to reduce their consumption of animal-based food (i.e., to become flexitarians) and thus eat more sustainably, since plant-based foods are generally regarded as more sustainable than animal-based ones. The novel products covered in this issue include plant-based and insect-based meat analogues [1,2,3], plant-based cheeses [4], plant-based dairy alternatives [5], and beverages made of pea protein as the main ingredient [6].

Several articles of this Special Issue also studied novel aspects that can make conventional plant-based foods contribute to sustainability and biodiversity. These studies addressed sensory properties and/or consumer acceptance of fortified lentils [7], extruded snacks made of legume flour and bran [8], white vs. brown rice [9], heritage cereals such as spelt and emmer wheat [10], and non-thermally processed fruit and vegetable products [11]. 

Consumers’ attitudes to various sustainable foods were investigated in several survey studies. Some of the studies focused on plant-based [2] or plant- and insect-based alternatives to meat [3], whereas others explored a wide range of sustainable foods and ingredients [12]. Modelling of data from consumer surveys was also used to study purchase intention for organic food in a discount setting [13] and the role of various factors on convenience food choice [14]. 

Consumers’ actual food choices/liking were also studied in experimental settings with foods to be tasted or eaten. In one study, consumers’ actual food choices and consumption were studied in an experimental lunch buffet in a multisensory environment [15]. Another study explored the impact of a “Mountain pasture product” claim on liking for cheese [16].

Minimizing food waste is another means to increase the sustainability of a food system. This aspect was addressed in studies on the acceptance of suboptimal citrus fruits [17] and unexploited, low-commercial-value fish species [18].

Topics of the articles in this Special Issue extend from foods to food packaging. One study explored consumers’ perspectives on sustainable paper-based packaging in a qualitative study using focus groups [19], whereas another study investigated sensory characteristics and consumer preferences of the conventional vs. sustainable packaging [20].

A wide variety of research techniques were applied in the studies. Of the 18 original articles, nine (50%) reported studies that included sensory analysis (i.e., at least one sense was used to evaluate the samples) [5,6,7,8,9,15,16,18,20], seven (~40%) were survey studies based on paper-and-pencil or online questionnaires [2,3,10,12,13,14,17], and two (~10%) used qualitative focus group interview approaches [11,19]. Furthermore, of the nine studies that included sensory analysis, two studies exclusively applied analytical sensory evaluation techniques using trained or semi-trained sensory panels [6,18], six studies employed hedonic tests to assess acceptability using non-trained panels (consumers) [5,7,8,9,15,16], while only one study used both [20]. Methods used in the studies demonstrate that the usual techniques of sensory and consumer research are also applicable to research on sustainable foods.

Analytical sensory techniques were used in studies that aimed to characterize the sensory properties of the samples as objectively as possible. Cosson et al. [6] compared three different sensory profiling methods (static block profiling, mono-intake temporal dominance of sensations (TDS) profiling, and multi-intake TDS profiling) for studying pea-protein-based beverages, especially for their beany, bitter, and astringent notes. Their results showed that the different profiling methods provided complementary information on the sensory properties of the beverages. Of the used methods, the multi-intake TDS profiling resembled real-life consumption and thus could provide additional information about how consumers perceive foods.

Silva et al. [18] studied sensory properties of unexploited fish species from the Portuguese coast during a year using check-all-that-apply (CATA) methods tailored to each species. The authors found seasonal influence on sensory attributes in four out of the five studied species and made conclusions on what time of the year would be most favorable for catching a specific fish species in sensory quality’s point of view.

Hedonic sensory tests were used in several studies. Oduro et al. [5] studied liking for a set of different plant-based milk analogues blending three plant beverages. They concluded that the multi-blend approach can be useful for improving sensory appeal and nutrient profiles as well as reducing over-reliance on a single plant material.

The fortification of plant-based foods for some nutrients may be beneficial for followers of vegetarian diets. Podder et al. [7] studied the effects of fortification with iron and zinc on liking for red and yellow lentils as uncooked and cooked among lentil consumers in Bangladesh, where the consumption of lentils is high. The authors concluded that, in general, the fortification decreased liking for the uncooked lentils, but not the cooked ones.

Proserpio et al. [8] studied liking for extruded snacks prepared with different ratios of pea and chickpea flours/brans blended with rice flour. In addition to hedonic value (measured using the Labeled Affective Scale, LAM) the consumer panel evaluated the samples using a CATA questionnaire with 23 sensory attributes. Using the combined data, the authors were able to conduct a penalty-lift analysis and show which sensory attributes significantly influenced overall liking. Moreover, the authors found that food neophobia was associated with lower liking for the novel snack products, particularly in women.

Gondal et al. [9] used a nine-point hedonic scale and just-about-right (JAR) scale to study consumer acceptability of brown and white rice varieties. The authors found that white rice varieties were preferred over their brown counterparts and that texture was the most important sensory attribute explaining the differences in liking.

Hoppu et al. [15] applied a sophisticated multisensory experimental setting for a lunch buffet to study effects of the eating environment to amount of food intake and emotions evoked. Compared to the control condition, the multisensory eating environment was rated as more pleasant and evoked more positive emotions, while no difference in food intake was found between the conditions.

Endrizzi et al. [16] studied the impact of external information, specifically a product claim “Mountain pasture product”, on the overall liking for tasted cheeses (nine-point hedonic scale). The authors found that the effect of the labeling information on the liking was positive and associated with consumers’ positive opinions with mountain pasture practices.

Lignou and Oloyede [20] used both analytical (trained panel) and hedonic (consumers) sensory analysis in their study on food packages. They employed several methods to study the sensory profile and consumer acceptability of sustainable paper-based packaging for two product categories (biscuit and meat). Both categories studied included a conventional plastic package and two or three paper-based prototypes. The authors concluded that while consumers were open to sustainable propositions, the design and size of the package were more important factors influencing consumer choice than the sustainable character of the packaging material.

Survey techniques were applied in several studies. These studies focused on revealing consumers’ attitudes and responses to foods using questionnaires (without tasting). Wendin et al. [10] conducted an online survey to investigate different consumer groups’ awareness, attitudes, and preferences toward heritage cereals such as spelt and emmer wheat in Sweden. Almost all respondents were aware of spelt, whereas the other heritage cereals (e.g., einkorn, emmer, Oland and Kamut wheat) were known by less than a half of the participants. Nevertheless, over 90% of the respondents expressed willingness to purchase bread made of heritage cereal.

Knaapila et al. [2] investigated millennials’ attitudes toward plant-based meat alternatives using an online survey in Finland. The authors classified the respondents to six consumer segments based on the hedonic tone of their first associations to meat and plant-based meat alternatives. While the extreme segments strongly preferred either meat or alternatives to meat, the middle segments had positive or neutral attitude to both. These segments were concluded to be flexitarians or prospective flexitarians and the best targets for future interventions designed to reduce meat consumption.

De Koning et al. [3] conducted a large survey on consumers’ attitudes toward and willingness to try and buy plant- and insect-based proteins in nine countries (Brazil, China, Dominican Republic, France, the Netherlands, New Zealand, Spain, the UK, and the USA, totaling 3091 responses) and analyzed the data using structural equation modelling (SEM). They concluded that behavioral intentions towards meat alternatives are inhibited by food neophobia but augmented by the perceived suitability and benefits of the protein (such as environmental impact, healthiness, nutritional importance, and sensory attributes).

Lundén et al. [12] conducted two online surveys to reveal consumers’ perspectives on a variety of novel, and partly traditional but marginally utilized, ingredients and foods in Finland. The results showed that plant-based ingredients are preferred over raw materials of animal origin, including insects. The authors concluded that Finnish consumers are not ready to adopt insects into their diet and that consumers need more knowledge and experience on cultivated meat and 3D food to accept them in their daily diets.

Katt and Meixner [13] ran a survey in the USA to examine the factors that influence discount grocery shoppers’ purchase intention for organic food, that is, usually premium priced compared to non-organic options. This study also employed SEM for data analysis. The results indicated that while price consciousness exhibited a negative relationship with the purchase intention, the impact of environmental concern, health consciousness, and hedonic shopping value was greater on the purchase intention of organic food than that of price consciousness (even in the discount setting).

Imtiyaz et al. [14] investigated the extent to which sensory appeal, nutritional quality, safety, and health determinants influence purchase intention, consumption, and satisfaction of consumers towards convenience food in India. Here, a purposive sampling method was used to recruit consumers of convenience foods. SEM was again used for data analysis. The authors concluded that, in emerging economies such as India, consumers give more importance to sensory appeal as compared with quality, safety, and health attributes during the purchase and consumption of convenience food.

Huang et al. [17] addressed an interesting question on consumer preferences for suboptimal foods in Taiwan. The authors studied effects of appearance, freshness (harvesting/packaging date), certification, and price discount on preferred choice of citrus fruit (ponkan, *Citrus poonensis*). Of the suboptimal citrus fruit certification attributes, the most important was the freshness indicator, followed by appearance, traceability certifications, price discounts, and finally size. That is, consumers were willing to compromise with fruit size but not with appearance or freshness. It would be interesting to see whether the same applies for other products or populations.

Focus group interviews (qualitative approach) were used in two studies [11,19]. Song et al. [11] investigated consumers’ perception and attitudes towards non-thermally processed fruit and vegetable products using focus groups (total 94 participants) in six European countries (Denmark, Germany, Italy, Serbia, Spain, and the Netherlands). They concluded that due to a lack of knowledge and trustworthy information sources, consumers had difficulties in assessing relevant benefits and risks in non-thermally processed fruit and vegetable products. The authors also recommended targeted communication (especially for middle-aged consumers) that could explicitly and efficiently reveal benefits and risks.

Oloyede and Lignou [19] conducted a qualitative study investigating consumers’ expectations and opinions of sustainable paper-based packaging materials using focus groups (total 60 participants) in the UK. The authors concluded that while the participants were concerned about the negative impact of the unsustainable packages on the environment, price and quality remained the key driving forces for consumers’ purchase intent—consumers may not be willing to pay more for a sustainable package.

Two review articles were published in this Special Issue. Fiorentini et al. [1] reviewed 14 studies on sensory properties and sensory-based consumer acceptance of plant-based meat analogues (12 studies) and meat extenders (2 studies). The authors found that, in terms of increasing consumer acceptance, studies have focused on ingredients and processing methods to improve especially the color, flavor, and texture of meat analogs. Regarding methodology, Fiorentini et al. stated what is generally applicable not only to meat analogs but all foods: “A combination of hedonic testing and descriptive analysis provides a more holistic understanding and an ideal approach to evaluate the sensory profile of meat analogs while also being able to identify the strategies to increase consumer acceptance of these novel foods”. However, only 1 of the 14 reviewed studies employed both analytic and hedonic sensory techniques.

Short et al. [4] made a systematic review on sensory studies on plant-based cheeses. The authors identified and reviewed 12 articles reporting sensory evaluation of (fully) plant-based cheese analogs. Most of the studied samples were soft (spreadable) and made of soy, either exclusively or blended with other plant-based ingredients. All of the studies applied a hedonic sensory method, while four of them also used a descriptive method. Short et al. noted that several studies had limitations in their methodology for sensory testing, such as a small number of participants and the use of trained panelists in hedonic testing. This review, especially the section Review of the Sensory Methods, provides helpful “dos and don’ts” for sensory evaluation of plant-based cheese analogs (and foods in general) for those who are not experts in sensory science but plan to use sensory techniques in their studies.

In conclusion, sensory and consumer research can support the development of food systems towards sustainability in many ways. Research in the field can help develop successful new products (such as meat and dairy analogs) and foster the use of existing sustainable options. Demand for more sustainable food drives change in food supply on the market, but consumer acceptance of new products cannot be taken for granted. Survey studies are essential for understanding various consumer segments in their needs, attitudes, and preferences. Sensory studies are needed to reveal the sensory properties of foods (qualitatively and quantitatively) and consumers’ hedonic responses to them. However, as Fiorentini et al. [1] and Short et al. [4] noted in their reviews, many previous sensory studies on meat and cheese analogs had limitations in their methodology, such as in the number of panelists, their training, and the statistical analysis of the data. Furthermore, both analytical and hedonic sensory techniques were employed jointly only in few studies. Sensory evaluation could also be utilized as combined with survey studies and chemical and physical analysis of the samples, to provide a more comprehensive understanding on factors influencing the sensory characteristics and consumer acceptance of the studied products. I hope that this Special Issue inspires readers for future studies in sensory and consumer research to support sustainability of the food system.
